# Blood culture status and mortality among patients with suspected community-acquired bacteremia: a population-based cohort study

**DOI:** 10.1186/1471-2334-11-139

**Published:** 2011-05-20

**Authors:** Mette Søgaard, Mette Nørgaard, Lars Pedersen, Henrik T Sørensen, Henrik C Schønheyder

**Affiliations:** 1 Department of Clinical Microbiology, Aalborg Hospital, Aarhus University Hospital, Aalborg, Denmark; 2 Department of Clinical Epidemiology, Clinical Institute, Aarhus University Hospital, Aarhus, Denmark; 3 Department of Epidemiology, Boston University, Boston, MA, USA

## Abstract

**Background:**

Comparison of mortality among patients with positive and negative blood cultures may indicate the contribution of bacteremia to mortality. This study (1) compared mortality among patients with community-acquired bacteremia with mortality among patients with negative blood cultures and (2) determined the effects of bacteremia type and comorbidity level on mortality among patients with positive blood cultures.

**Methods:**

This cohort study included 29,273 adults with blood cultures performed within the first 2 days following hospital admission to an internal medical ward in northern Denmark during 1995-2006. We computed product limit estimates and used Cox regression to compute adjusted mortality rate ratios (MRRs) within 0-2, 3-7, 8-30, and 31-180 days following admission for bacteremia patients compared to culture-negative patients.

**Results:**

Mortality in 2,648 bacteremic patients and 26,625 culture-negative patients was 4.8% vs. 2.0% 0-2 days after admission, 3.7% vs. 2.7% 3-7 days after admission, 5.6% vs. 5.1% 8-30 days after admission, and 9.7% vs. 8.7% 31-180 days after admission, corresponding to adjusted MRRs of 1.9 (95% confidence interval (CI): 1.6-2.2), 1.1 (95% CI: 0.9-1.5), 0.9 (95% CI: 0.8-1.1), and 1.0 (95% CI: 0.8-1.1), respectively. Mortality was higher among patients with Gram-positive (adjusted 0-2-day MRR 1.9, 95% CI: 1.6-2.2) and polymicrobial bacteremia (adjusted 0-2-day MRR 3.5, 95% CI: 2.2-5.5) than among patients with Gram-negative bacteremia (adjusted 0-2-day MRR 1.5, 95% CI 1.2-2.0). After the first 2 days, patients with Gram-negative bacteremia had the same risk of dying as culture-negative patients (adjusted MRR 0.8, 95% CI: 0.5-1.1). Only patients with polymicrobial bacteremia had increased mortality within 31-180 days following admission (adjusted MRR 1.3, 95% CI: 0.8-2.1) compared to culture-negative patients. The association between blood culture status and mortality did not differ substantially by level of comorbidity.

**Conclusions:**

Community-acquired bacteremia was associated with an increased risk of mortality in the first week of medical ward admission. Higher mortality among patients with Gram-positive and polymicrobial bacteremia compared with patients with Gram-negative bacteremia and negative cultures emphasizes the prognostic importance of these infections.

## Background

Community-acquired bacteremia is a serious condition with a hospitalization rate of approximately 80 per 100,000 person-years [1] and a 30-day mortality greater than 15% [2]. Mortality results from complex interactions of factors including the infectious agent, host immune response, underlying disorders, diagnostic procedures, and medical interventions. These factors and their effects can be difficult to untangle [3,4].

A comparison of mortality between patients with positive blood cultures and patients with negative blood cultures may indicate the contribution of bacteremia to mortality. Two hospital-based cohort studies have suggested that 30-day mortality was two- to three-fold higher among patients with bacteremia compared to patients with negative blood cultures [4,5], while 1-year mortality was similar between the two groups (mortality rate ratio (MRR) 1.3, 95% confidence interval (CI): 0.76-2.10) [5]. In contrast, an Israeli cohort study found that mortality was two-fold higher 6 months after admission among patients with bacteremia and remained elevated for up to 4 years, compared to patients with the same underlying conditions but no infectious diseases [3]. Two other cohort studies from France and Canada identified bacteremia as a predictor of in-hospital mortality in intensive care unit (ICU) patients with sepsis, severe sepsis, or septic shock, with reported relative risks of 1.6 [6] and 1.7 [7], respectively. To date, no study has focused on mortality in community-acquired bacteremia. Other limitations of earlier studies include lack of long-term follow-up [4,6,7], small sample size [5], and lack of adjustment for coexisting chronic diseases [4,7].

To compare mortality among patients with community-acquired bacteremia with that among patients with negative blood cultures, controlling for potential confounders, we conducted a cohort study involving hospitalized internal medicine patients who had one or more blood cultures performed within 2 days following admission. Our aims were (1) to examine the association between blood culture status, defined as positive (*i.e. *bacteremia) or negative blood cultures, and early mortality (within 0-2 and 3-7 days following hospital admission), short-term mortality (within 8-30 days), and long-term mortality (within 31-180 days); and (2) to determine the effects of bacteremia type and comorbidity level on mortality among patients with blood cultures.

## Materials and methods

### Study setting and population

**Figure 1:**
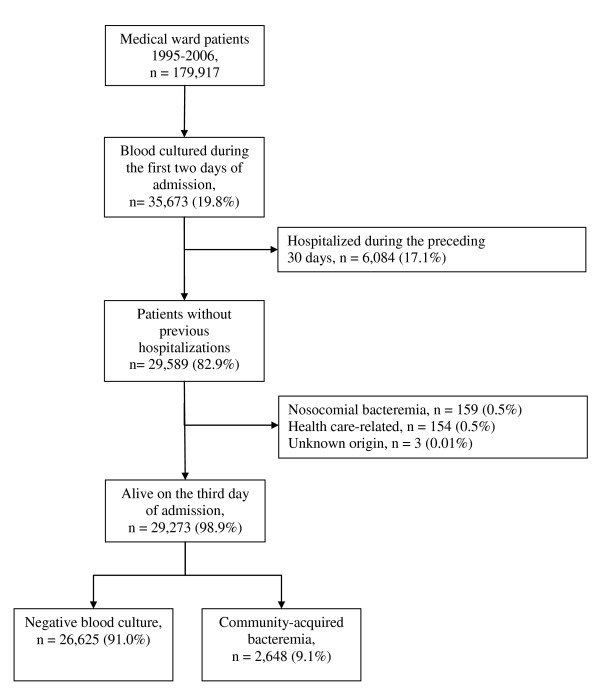
**Flow diagram of the study population**.

We conducted this population-based cohort study in northern Denmark (population ~500,000) between 1995 and 2006. Universal access to free tax-supported health care is provided in Denmark, and since 1968, each Danish resident has been assigned a civil registration number that permits unambiguous record linkage [8,9]. The study included all patients over age 15 years who had one or more blood cultures performed within 2 days of hospital admission to an internal medicine ward. The 17 medical departments in the region represented general internal medicine and the following medical specialties: endocrinology, gastroenterology, geriatrics, hematology, infectious diseases, nephrology, and pulmonary diseases. Four allied specialty departments included cardiology, medical oncology, neurology, and rheumatology. To be eligible, patients had to have had no previous blood cultures and no hospital contact during the preceding 30 days (Figure 1).

### Blood culture data

Blood culture data were obtained from the laboratory information system maintained by the Department of Clinical Microbiology, Aalborg Hospital. Two blood culture systems were used during the study period: the Colorbact system in 1995 (Statens Serum Institut, Copenhagen, Denmark) [10] and the BacT/Alert system (bioMérieux, Marcy l'Etoile, France) between 1996 and 2006. The nominal volume per blood culture was 20-22 mL for the Colorbact system and 28-32 mL for the BacT/Alert system. Positive blood cultures were identified at fixed times and examined immediately by a technician [11]. On the basis of microscopy results, a first telephone notification was made to the attending physicians; antibiotic treatment was adjusted if deemed necessary. As soon as the antibiotic susceptibility pattern of the isolate was obtained, a second contact was made either to confirm or adjust antibiotic treatment. Negative blood cultures were incubated for approximately 7 days and then a written report was sent to the attending physicians.

### Data on community-acquired bacteremia

We defined bacteremia as the presence of viable bacteria or fungi in the bloodstream, evidenced in blood cultures in which contamination had been ruled out [12]. We regarded coagulase-negative staphylococci, *Corynebacterium *spp., *Bacillus *spp., and *Propionibacterium acnes *as contaminants unless they were isolated from two or more separate blood culture sets [12,13]. Community-acquired bacteremia was defined as an infection present or incubating at the time of admission [14]; thus, we considered positive blood cultures obtained within the first 2 days of admission as evidence of community-acquired bacteremia. Patients with a hospital stay in the 30 days prior to admission or who had regular hospital visits (*e.g. *for hemodialysis or chemotherapy) were categorized as having health care-associated bacteremia and were not included in the study [15,16].

Patients with community-acquired bacteremia were identified from the North Denmark Region Bacteremia Research Database [12]. This database has recorded all episodes of bacteremia since 1992 at the time of the clinical episode. We categorized the bacteremia episodes as Gram-positive, Gram-negative, polymicrobial bacteremia, and fungemia. Polymicrobial bacteremia was defined as an episode with more than one clinically important blood culture isolate detected within 48 hours unless a new focus of infection had become apparent [12,17].

### Data on comorbidity and marital status

Comorbidity was classified according to the Charlson Comorbidity Index (CCI) [18]. We computed CCI scores based on all previous diagnoses recorded in the Danish National Patient Registry, which has covered all Danish hospitals since 1977. The diagnoses were coded by physicians at discharge according to the *International Classification of Diseases*, 8th revision (ICD-8) until the end of 1993 and with the 10th revision (ICD-10) thereafter. We defined three levels of comorbidity: low (score of 0), medium (scores of 1-2), and high (score of >2) [19]. As a marker of social status [20,21], we obtained information from the Civil Registration System on marital status (married, never married, divorced, or widowed) on the date of the blood culture.

### Statistical analysis

Data on mortality among study subjects were obtained from the Civil Registration System [8,9]. Follow-up started on the day of admission and extended for 180 days or until death or migration, whichever occurred first. We constructed Kaplan-Meier curves and categorized follow-up time into four intervals: early mortality (within the first 0-2 days and at 3-7 days following admission), short-term mortality (within 8-30 days following admission), and long-term mortality (within 31-180 days following admission). To compare mortality among patients with positive vs. negative blood cultures, we computed product limit estimates and used Cox regression analysis to compute MRRs for each time period with 95% confidence intervals (CI). The indications for blood cultures may have changed over time, and all models included adjustment for calendar period (1995-1998, 1999-2002, and 2003-2006) as well as age (15-39, 40-59, 60-79, ≥ 80 years), gender, marital status, and level of comorbidity.

Because some cultures might have been performed to rule out bacteremia rather than because of a clinical suspicion of bacteremia, we conducted an analysis restricted to patients with a primary or secondary infectious disease diagnosis in the hospital registry (please see additional file 1: Hospital diagnoses and ICD-10 codes). We assumed that these patients had a high degree of clinical suspicion of bacteremia. We also categorized bacteremia into different types and stratified the analysis by comorbidity level. We graphically assessed the assumption of proportional hazards in the Cox model and found it appropriate for each follow-up interval. Statistical analyses were performed using Stata Statistical Software v. 9.2 (Stata Corp., College Station, TX, USA). The study was approved by the Danish Data Protection Agency (http://www.datatilsynet.dk/english/, record no. 2006-41-7413). Data were obtained from Danish registries, which are openly available to researchers, and their use does not require informed consent. Records from the registries were linked by means of each patient's unique civil registration number, and after record linkage, the data were de-identified.

## Results

During the study period, we identified 179,917 patients admitted to internal medicine departments, of whom 35,673 (19.8%) had at least one blood culture performed within the first 2 days following admission. After exclusion of patients hospitalized within the preceding 30 days (n = 6,084, 17.1%) and patients with non-community-acquired bacteremia (n = 316, 1.1%), our study population consisted of 29,273 internal medicine patients. Of these, 26,625 (91.0%) had negative blood cultures and 2,648 (9.1%) had community-acquired bacteremia (Figure 1). Table 1 presents the hospital registry diagnoses of patients in the study population.

**Table 1:** Hospital diagnoses of 29,273 inpatients who were not hospitalized in the preceding 30 days and who had blood cultures taken within the first 2 days of hospital admission‡.

	Blood culture	
	
Hospital registry diagnosis	Negative No. (%)	Positive No. (%)
Total	26,625	2,648
Acute or subacute infectious diseases		
Primary discharge diagnosis	11,481 (43.1)	2,010 (75.9)
Primary or secondary discharge diagnosis	13,928 (52.3)	2,256 (85.2)
Pneumonia	7,107 (26.7)	678 (25.6)
Urinary tract infection	2,567 (9.6)	534 (20.2)
Intestinal infectious disease	1,034 (3.9)	77 (2.9)
Fever of unknown origin	768 (2.9)	20 (0.8)
Neoplasm	1,124 (4.2)	42 (1.6)
Anemia	283 (1.1)	9 (0.3)
Diabetes	406 (1.5)	12 (0.5)
Neurological or psychiatric disease	605 (2.3)	11 (0.4)
Cardiovascular disease	2,836 (10.7)	66 (2.5)
Respiratory disease	1,772 (6.7)	28 (1.1)
Gastrointestinal disease	1,062 (4.0)	87 (3.3)
Skin, connective tissue, or musculoskeletal disease	740 (2.8)	21 (0.8)
Renal and urinary tract disease	271 (1.0)	23 (0.8)
Symptoms without a specific diagnosis*	2,158 (8.3)	54 (2.1)
Injury and poisoning from medicines	317 (1.2)	6 (0.2)
Other^†^	309 (1.2)	8 (0.3)

‡ For information about codes please see additional file 1: Hospital diagnoses and ICD-10 codes)

*Includes patients under evaluation for suspected diseases and conditions (~50%), patients with dehydration (~12%), patients with disorientation, dizziness, headache, syncope or collapse (~12%), and patients with pain, nausea, or vomiting.

^† ^Includes endocrine and metabolic disorders (other than diabetes), as well as diseases of the eye and ear.

### Patient characteristics

Patients with bacteremia were older (median age, 73; interquartile range (IQR): 59-82 years) than patients with negative blood cultures (median age, 68; IQR: 50-79 years). Compared with culture-negative patients, patients with bacteremia had slightly higher prevalences of congestive heart failure, peripheral vascular disease, peptic ulcer disease, and diabetes. Patients with negative cultures had a higher prevalence of chronic pulmonary disease (15.0% vs. 10.7% in patients with bacteremia) (Table 2). Among patients with community-acquired bacteremia, 53.5% had medium or high comorbidity scores, compared with 50.1% of patients with negative cultures.

**Table 2:** Descriptive characteristics of 29,273 inpatients with one or more blood cultures taken within the first 2 days following hospital admission.

	Blood culture	
	
	Negative No. (%)	Positive No. (%)
Total	26,625 (91.0)	2,648 (9.1)
Time of blood culture		
Day of admission	20,158 (75.7)	2,228 (84.1)
First day following admission	4,921 (18.5)	368 (13.9)
Second day following admission	1,546 (5.8)	52 (2.0)
Calendar period		
1995-1998	8,703 (32.7)	925 (35.0)
1999-2002	8,402 (31.6)	838 (31.7)
2003-2006	9,520 (35.8)	885 (33.4)
Marital status		
Married	12,349 (46.4)	1,204 (45.5)
Never married	5,097 (19.1)	345 (13.0)
Divorced or widowed	8,902 (33.4)	1061 (40.1)
Unknown	277 (1.0)	38 (1.4)
Gender		
Male	13,543 (50.9)	1,216 (45.9)
Female	13,082 (49.1)	1,432 (54.1)
Age group (years)		
15-39	4,405 (16.5)	206 (7.8)
40-59	5,353 (20.1)	481 (18.2)
60-79	10,835 (40.7)	1,168 (44.1)
80 and older	6,032 (22.7)	793 (30.0)
Comorbidity		
Previous myocardial infarction	1,720 (6.5)	182 (6.9)
Congestive heart failure	1,934 (7.3)	246 (9.3)
Peripheral vascular disease	1,451 (5.5)	181 (6.8)
Cerebrovascular disease	2,710 (10.2)	283 (10.7)
Dementia	434 (1.6)	41 (1.6)
Hemiplegia	121 (0.5)	6 (0.2)
Chronic pulmonary disease	3,992 (15.0)	284 (10.7)
Connective tissue disease	1,098 (4.1)	136 (5.1)
Peptic ulcer disease	1,794 (6.7)	211 (8.0)
Mild liver disease	355 (1.3)	62 (2.3)
Moderate to severe liver disease	84 (0.3)	20 (0.8)
Diabetes without end-stage organ damage	1,856 (7.0)	255 (9.6)
Diabetes with end-stage organ damage	888 (3.3)	103 (3.9)
Moderate to severe renal disease	647 (2.4)	73 (2.8)
Solid cancer	2,752 (10.6)	293 (11.6)
Metastatic solid cancer	376 (1.4)	50 (1.9)
Leukemia	172 (0.7)	16 (0.6)
Lymphoma	316 (1.2)	26 (1.0)
AIDS	28 (0.1)	3 (0.1)
Charlson Comorbidity Index Score		
Low score (0)	13,297 (49.9)	1,231 (46.5)
Medium score (1-2)	9,654 (36.3)	993 (37.5)
High score (>2)	3,674 (13.8)	424 (16.0)

Of patients with bacteremia, 84.1% had their first positive culture taken on the day of admission. In comparison, 75.7% of the negative cultures were taken on the day of admission (Table 2). Among the 2,648 bacteremia patients, a total of 1,145 (43.2%) had Gram-positive bacteremia while 1,340 (50.6%) had Gram-negative bacteremia, 160 (6.0%) had polymicrobial bacteremia, and 3 had fungemia (caused by *Candida albicans*, *Candida glabrata*, and *Saccharomyces cerevisiae*, respectively). *Streptococcus pneumoniae *accounted for 54.7% of the Gram-positive pathogens, *Staphylococcus aureus *accounted for 17.0%, and beta-hemolytic streptococci for 13.3%. The Gram-negative bacteremias were predominantly Enterobacteriacae (90.0%). *Escherichia coli *accounted for 79.4% of all Gram-negative bacteremias.

### Early and short-term mortality

**Figure 2:**
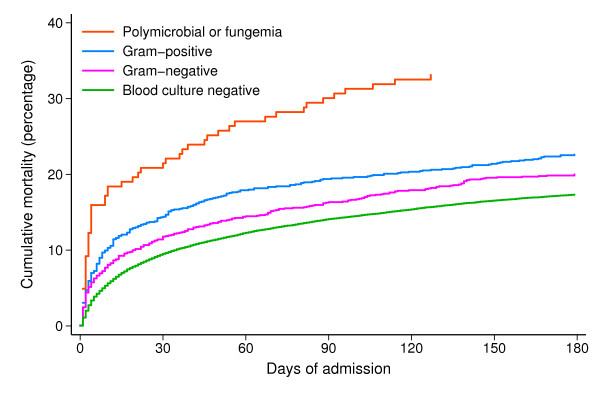
**Mortality curves for the 29,273 patients by blood culture result and type of bacteremia**.

Figure 2 shows mortality curves for patients during the 180 days following admission, stratified by blood culture result and type of bacteremia. The overall mortality within the first 2 days following admission was 2.0% in patients with negative cultures and 4.8% in patients with bacteremia (adjusted 0-2-day MRR 1.9, 95% CI 1.6-2.2) (Table 3). Highest 0-2-day mortality was seen for patients with polymicrobial bacteremia (9.2%, adjusted MRR 3.5, 95% CI 2.2-5.5). For patients with Gram-positive bacteremia and Gram-negative bacteremia, the 0-2-day mortality was 4.7% (adjusted MRR 2.1, 95% CI 1.5-2.7) and 4.4% (adjusted MRR 1.5, 95% CI 1.2-2.0), respectively.

**Table 3:** Crude and adjusted risk of death within 0-2 days following hospital admission among inpatients who had one or more blood cultures taken within 2 days of hospital admission.

Blood culture status	n	0-2 days following admission		
		
		Mortality, % (95% CI)	Crude MRR (95% CI)	Adj. MRR* (95% CI)
Negative	26,625	2.0 (1.9-2.2)	1.0 (ref)	1.0 (ref)
Positive	2,648	4.8 (4.1-5.7)	2.2 (1.9-2.6)	1.9 (1.6-2.2)
Gram-positive	1,145	4.7 (3.6-6.1)	2.2 (1.7-2.8)	2.1 (1.6-2.7)
Gram-negative	1,340	4.4 (3.4-5.7)	1.9 (1.5-2.4)	1.5 (1.2-2.0)
Polymicrobial	160	9.2 (5.7-14.8)	4.6 (3.0-7.2)	3.5 (2.2-5.5)

*Adjusted for age, gender, level of comorbidity, marital status, and calendar period.

The 3-7-day mortality rate was 3.7% in patients with bacteremia and 2.7% in patients with negative cultures, resulting in an adjusted MRR of 1.1 (95% CI: 0.9-1.5). Patients with polymicrobial bacteremia had the highest mortality rates (3-7-day mortality = 7.4%), followed by patients with Gram-positive bacteremia (3-7-day mortality = 4.5%). Using culture-negative patients as the reference, the adjusted 3-7-day MRR was 1.4 (95% CI: 0.6-3.1) for patients with polymicrobial bacteremia, 1.7 (95% CI: 1.2-2.3) for patients with Gram-positive bacteremia, and 0.8 (95% CI: 0.5-1.1) for patients with Gram-negative bacteremia (Table 4).

**Table 4:** Crude and adjusted risk of death within 3-7, 8-30, and 31-180 days following hospital admission among inpatients with one or more blood cultures taken within 2 days of hospital admission.

Blood culture status	3-7** days following admission			8-30 days following admission^†^			31-180 days following admission^‡^		
	
	Mortality, % (95% CI)	Crude MRR (95% CI)	Adj. MRR* (95% CI)	Mortality, % (95% CI)	Crude MRR (95% CI)	Adj. MRR* (95% CI)	Mortality, % (95% CI)	Crude MRR (95% CI)	Adj. MRR* (95% CI)
Negative	2.7 (2.5-2.9)	1.0 (ref)	1.0 (ref)	5.1 (4.9-5.4)	1.0 (ref)	1.0 (ref)	8.7 (8.3-9.0)	1.0 (ref)	1.0 (ref)
Positive	3.7 (3.1-4.6)	1.3 (1.1-1.7)	1.1 (0.9-1.5)	5.6 (4.8-6.6)	1.1 (0.9-1.3)	0.9 (0.8-1.1)	9.7 (8.6-11.0)	1.1 (1.0-1.3)	1.0 (0.8-1.1)
Gram-positive	4.5 (3.4-5.9)	1.7 (1.3-2.4)	1.7 (1.2-2.3)	6.0 (4.7-7.6)	1.1 (0.9-1.5)	1.1 (0.8-1.4)	9.6 (7.9-11.6)	1.1 (0.9-1.4)	1.0 (0.8-1.3)
Gram-negative	2.7 (1.9-3.7)	1.0 (0.7-1.4)	0.8 (0.5-1.1)	5.2 (4.1-6.6)	1.0 (0.8-1.3)	0.8 (0.6-1.0)	9.3 (7.8-11.1)	1.1 (0.9-1.3)	0.9 (0.7-1.0)
Polymicrobial	7.4 (4.2-13.0)	1.9 (0.8-4.2)	1.4 (0.6-3.1)	6.6 (3.5-12.2)	1.5 (0.8-2.8)	1.1 (0.6-2.1)	14.8 (9.7-22.3)	1.7 (1.1-2.8)	1.3 (0.8-2.1)

*Adjusted for age, gender, level of comorbidity, marital status, and calendar period.

** For patients alive on day 3

^†^For patients alive on day 8.

^‡^For patients alive on day 31.

Death during days 8-30 following admission occurred in 5.6% of patients with bacteremia vs. 5.1% of patients with negative cultures, equivalent to an adjusted MRR of 0.9 (95% CI: 0.8-1.1). The association between blood culture status and mortality changed little with stratification by type of bacteremia (Table 4). However, the estimates for patients with polymicrobial bacteremia were imprecise.

### Long-term mortality

For all patients alive on day 30 after admission, follow-up lasted for 180 days. During days 31-180, 9.7% of patients with bacteremia died compared with 8.7% of patients with negative cultures (Table 4). The corresponding adjusted MRR was 1.0 (95% CI: 0.8-1.1). Only polymicrobial bacteremia was associated with increased long-term mortality compared to negative cultures (adjusted MRR 1.3, 95% CI: 0.8-2.1).

As Table 5 shows, mortality increased with level of comorbidity. Within each level of comorbidity, community-acquired bacteremia was associated with increased early mortality. In an analysis restricted to patients with a primary or secondary infectious disease diagnosis, we found that mortality in bacteremia patients compared with culture-negative patients was 4.4% vs. 1.7% after 0-2 days, 3.4% vs. 2.2% after 3-7 days, 5.1% vs. 4.2% after 8-30 days, and 8.8% vs. 6.7% after 31-180 days, equivalent to adjusted MRRs of 2.0 within 0-2 days, 1.3 (95% CI: 1.0-2.7) within 3-7 days, 1.0 (95% CI: 0.8-1.3) within 8-30 days, and 1.1 (95% CI: 0.9-1.3) within 31-180 days following hospital admission, respectively.

**Table 5:** Crude and adjusted risk of death within 0-2, 3-7, 8-30, and 31-180 days following hospital admission among by level of comorbidity.

		0-2 days following admission		3-7 days following admission**		**8-30 days following admission**^†^		**31-180 days following admission**^‡^	
		
Comorbidity	Blood culture status	Mortality, % (95% CI)	Adjusted MRR* (95% CI)	Mortality, % (95% CI)	Adjusted MRR (95% CI)	Mortality, % (95% CI)	Adjusted MRR (95% CI)	Mortality, % (95% CI)	Adjusted MRR (95% CI)
Low	Negative	1.4 (1.2-1.6)	1.0 (ref)	1.9 (1.6-2.1)	1.0 (ref)	3.4 (3.1-3.7)	1.0 (ref)	5.0 (4.6-5.4)	1.0 (ref)
	Positive	3.7 (2.8-5.0)	1.8 (1.4-2.5)	2.4 (1.6-3.4)	0.9 (0.6-1.4)	4.0 (3.0-5.3)	0.9 (0.7-1.2)	6.8 (5.4-8.4)	1.1 (0.8-1.4)
									
Medium	Negative	2.3 (2.0-2.6)	1.0 (ref)	3.1 (2.8-3.5)	1.0 (ref)	6.1 (5.6-6.6)	1.0 (ref)	10.7 (10.0-11.3)	1.0 (ref)
	Positive	5.3 (4.1-6.9)	1.9 (1.4-2.4)	4.3 (3.1-5.8)	1.4 (1.0-1.9)	5.9 (4.5-7.6)	0.9 (0.7-1.2)	10.9 (9.0-13.2)	0.9 (0.7-1.2)
									
High	Negative	3.4 (2.9-4.1)	1.0 (ref)	4.5 (3.9-5.3)	1.0 (ref)	9.2 (8.3-10.2)	1.0 (ref)	18.1 (16.8-19.5)	1.0 (ref)
	Positive	6.8 (4.8-9.7)	2.0 (1.5-2.9)	6.6 (4.5-9.5)	1.1 (0.7-1.9)	10.0 (7.4-13.6)	1.0 (0.7-1.5)	16.9 (13.3-21.4)	0.9 (0.7-1.2)

*Adjusted for age, gender, marital status, and calendar period.

** For patients alive on day 3.

^†^For patients alive on day 8.

^‡^For patients alive on day 31.

## Discussion

In this study of 29,273 patients hospitalized in internal medicine wards, we found a two-fold higher mortality during the first 2 days following admission among patients with community-acquired bacteremia compared with patients with negative blood cultures. Thereafter, mortality among patients with bacteremia was only slightly higher than among patients with negative cultures. Stratifying by type of bacteremia showed that mortality was highest during the first week after admission among patients with Gram-positive and polymicrobial bacteremia, but patients with polymicrobial bacteremia had an increased mortality for at least 180 days. After the first 2 days following admission, mortality in patients with Gram-negative bacteremia was similar to patients with negative blood cultures. The level of comorbidity did not greatly influence the association between blood culture status and mortality.

Although our use of data from population-based registries with complete follow-up and the universal health care coverage in Denmark minimized selection bias, we have to consider potential weaknesses when interpreting our results. We had no data on the indications for performing blood cultures. Therefore, to define a population of patients with suspected community-acquired bacteremia, we restricted the study to patients with blood cultures performed within 2 days of admission to an internal medicine ward and with no hospital contact during the preceding 30 days. Because we defined our condition of community-acquired bacteremia as any positive blood culture obtained within the first 2 days following hospital admission, the patient's exposure status could vary over the first 2 days of admission. Thus, patients with negative blood cultures who died shortly after admission may have had bacteremia that was undiagnosed. Compared with patients with bacteremia, culture-negative patients were less likely to have their blood cultures taken on the day of admission, and as a result, our relative estimates of mortality in the first 2 days following admission may have been biased because of misclassification of exposure status. Some patients with negative cultures may have had undetected bacteremia, particularly if they had received antimicrobial therapy [22]; this lack of detection could have led to underestimation of bacteremia-related mortality and consequently to more conservative estimates of relative mortality. Because we used administrative data lacking clinical detail, we had no information for evaluating a patient's clinical state at the time of blood culturing or about potential empirical antibiotic treatment in patients with negative blood cultures, which could have contributed to a favourable prognosis.

Measurement of disease severity in patients with community-acquired bacteremia poses a particular challenge because it is difficult to assess disease severity at the optimal time point immediately before the onset of bacteremia [23]. Therefore, some of the clinical data used to calculate acute severity scores and the clinical presentation actually may be influenced by the bacteremia. Finally, we computed the CCI based on all previous diagnoses recorded in the Danish National Patient Registry. The CCI is widely used but cannot assess the existence of comorbidity as accurately as clinical data [24]. Thus, inaccuracy of discharge data and diagnoses not included in the index may have reduced our ability to estimate comorbidity completely, and misclassification may have led to residual confounding that could have affected our mortality estimates. The validity of discharge diagnoses registered in the Danish National Patient Registry is variable but is generally high for the diseases included in the CCI [25]. In any case, the results of blood cultures obtained during a current admission were unlikely to have affected the accuracy of the diagnoses from previous admissions. Thus, any misclassification should bias the observed mortality estimates toward unity.

By focusing on patients with community-acquired bacteremia and stratifying by type of bacteremia, our data extend previous studies comparing the prognosis of patients with positive and negative blood cultures [4,5]. A Canadian study found that patients with bacteremia had higher in-hospital mortality in the 30 days after a positive blood culture than patients with a negative blood culture (27.3% vs. 7.3%) [4]. In line with that study, we observed the highest mortality among patients with polymicrobial bacteremia; however, the Canadian study, unlike the current work, identified no difference in 30-day mortality between bacteremia caused by Gram-positive cocci and Gram-negative bacilli. Nonetheless, our result of a better prognosis with Gram-negative bacteremia is corroborated by the finding of a low mortality from *E. coli *bacteremia with urinary tract foci in the Canadian study. In a single hospital study from the US, Bates et al. [5] reported a higher short-term mortality (adjusted 30-day MRR 2.3, 95% CI: 1.2-4.4) but less-affected long-term mortality (adjusted 1-year MRR 1.3, 95% CI: 0.76-2.1) among 142 bacteremia patients compared with 142 patients with negative blood cultures (matched by age, gender, severity of underlying disease, and the presence of major comorbidity). Our study results are in accord with this result and further suggest that the increased mortality associated with community-acquired bacteremia occurs predominantly within the first days after admission. Conversely, an Israeli study reported an increased long-term mortality in patients with bacteremia when compared to controls without infectious diseases, matched by age, gender, underlying diseases, hospital department, and admission date (43% vs. 19% 180-day mortality rate) [3]. The use of different comparison cohorts most likely explains this discrepancy.

Recently, Laupland et al. [7] reported that bacteremia was associated with a 60% increase in in-hospital mortality (crude OR 1.6, 95% CI: 1.1-2.2) among Canadian ICU patients with sepsis. When they controlled for variables that reflected the acute systemic response to infection, the adjusted OR was 1.1 (95% CI: 0.7-1.8), suggesting that the effect of bacteremia is mediated to a large extent by the severity of the acute systemic inflammatory response. Similarly, a study based on data from 170 French ICUs showed that bacteremia in patients with severe sepsis or septic shock was associated with mortality within 3 days of ICU admission (adjusted OR 1.7, 95% CI: 1.1-2.8) but not at 28 days after admission [6]. We used overall mortality as our outcome measure because of the difficulty of distinguishing the contributions to mortality of the septic process and of underlying disorders. However, the inclusion of patients with negative blood cultures as a reference in our study is important when interpreting our findings because we surmise that patients with negative cultures had presented with several essential signs of sepsis, not only pyrexia. Our MRRs therefore may reflect the effect of bacteremia per se on mortality. Although underlying comorbidities may have influenced the physician's decision to obtain blood cultures, the association between blood culture status and mortality remained robust in analyses restricted to patients with infectious disease registry diagnoses and in different comorbidity strata.

## Conclusions

Community-acquired bacteremia was associated with a markedly increased mortality in the first 2 days following admission to a hospital internal medicine ward compared with patients with negative cultures. However, the excess mortality declined notably after that time, emphasizing the clinical importance of prevention, early detection, and effective empirical antibiotic treatment for suspected but not yet proven bacteremia. Traditionally, Gram-negative bacteria have received the most attention because of their role in endotoxin-induced shock. Our finding of higher mortality among patients with Gram-positive and polymicrobial bacteremia is clinically important and suggests that identification of these types of infections requires greater attention.

## Competing interests

The authors declare that they have no competing interests.

## Authors' contributions

MS designed the study, performed statistical analysis, analyzed and interpreted the data, and wrote the manuscript. MN conceived and designed the study, analyzed and interpreted data, and critically revised the manuscript. LP performed statistical analysis and contributed to the design of the study and preparation of the paper. HTS and HCS conceived and designed the study, analyzed and interpreted the data, and critically revised the manuscript. All authors read and approved the final manuscript.

## Pre-publication history

The pre-publication history for this paper can be accessed here:

http://www.biomedcentral.com/1471-2334/11/139/prepub

## Supplementary Material

Additional file 1 **Hospital diagnoses and ICD-10 codes**. Hospital diagnoses and corresponding ICD-10 codes of 29,273 inpatients who were not hospitalized in the preceding 30 days and who had blood cultures taken within the first 2 days of hospital admission. Click here for file
