# Supplementary material for: Blood culture status and mortality among patients with suspected community-acquired bacteremia: a population-based cohort study
Source: BMC Infect Dis. 2011 May 20;11:139. doi: 10.1186/1471-2334-11-139 (PMC3128048; doi:10.1186/1471-2334-11-139)
Supplement: Additional file 1 — Hospital diagnoses and ICD-10 codes. Hospital diagnoses and corresponding ICD-10 codes of 29,273 inpatients who were not hospitalized in the preceding 30 days and who had blood cultures taken within the first 2 days of hospital admission. [file 1471-2334-11-139-S1.DOC]

**Additional file 1.** Hospital diagnoses and ICD-10 codes.

| Hospital registry diagnosis | ICD-10 codes |
| --- | --- |
| Acute or subacute infectious diseases | A00-B99, G00-G02, I32, I33, I41, J00-J06, J10-J18, J20-J22, J36, J85-J86, K65, L00-L03, L080, L088-L080, M00-M01, N10, N12, N30, or N39.0 |
| Pneumonia | J12-J18 |
| Urinary tract infection | N10, N12, N30.0, N30.9, N39.0 |
| Intestinal infectious disease | A00-A09 |
| Fever of unknown origin | R50 |
| Neoplasm | C00-C97 |
| Anemia | D50-D89 |
| Diabetes Mellitus | E10-E14 |
| Neurological or psychiatric disease | F00-F99, G00-G99 |
| Cardiovascular disease | I00-I99 |
| Respiratory disease | J00-J99 |
| Gastrointestinal disease | K00-K93 |
| Skin, connective tissue, or musculoskeletal disease | L00-L99, M00-M99, D86.3 |
| Renal and urinary tract disease | N00-N99 |
| Symptoms without a specific diagnosis | R00-R99, Z00-Z99, E86.9 |
| Injury and poisoning from medicines | S00-T98 |
| Other | H00-H95, D00-D48, E00-E07, E15-E90, V01-Y98 |
